# Startle-triggered responses indicate reticulospinal drive is larger for voluntary shoulder versus finger movements

**DOI:** 10.1038/s41598-023-33493-5

**Published:** 2023-04-21

**Authors:** Dana Maslovat, Cassandra M. Santangelo, Anthony N. Carlsen

**Affiliations:** School of Human Kinetics, University of Ottawa, 125 University Private, Ottawa, ON K1N 6N5 Canada

**Keywords:** Motor control, Neurophysiology

## Abstract

Recent primate studies have implicated a substantial role of reticulospinal pathways in the production of various voluntary movements. A novel way to assess the relative reticulospinal contributions in humans is through the use of a “StartReact” paradigm where a startling acoustic stimulus (SAS) is presented during a simple reaction time (RT) task. The StartReact response is characterized by short-latency triggering of a prepared response, which is attributed to increased reticulospinal drive associated with startle reflex activation. The current study used a StartReact protocol to examine differences in reticulospinal contributions between proximal and distal effectors by examining EMG onset latencies in lateral deltoid and first dorsal interosseous during bilateral shoulder or finger abduction. The magnitude of the StartReact effect, and thus relative reticulospinal drive, was quantified as the difference in RT between startle trials in which startle-reflex related EMG activation in the sternocleidomastoid (SCM) was present (SCM +) versus absent (SCM −). A significantly larger StartReact effect was observed for bilateral shoulder abduction versus bimanual finger abduction and a higher incidence of SCM + trials occurred in the proximal task. Additionally, both startle reflex and response-related EMG measures were larger on SCM + trials for the shoulder versus finger task. These results provide compelling novel evidence for increased reticulospinal activation in bilateral proximal upper-limb movements.

## Introduction

Reticulospinal pathways are known to be involved in human movement, yet traditionally these brainstem structures have been thought to predominantly contribute to the generation of gross actions using proximal muscles such as those used in locomotion and postural adjustments^1,2^. However, it is becoming increasingly clear the reticulospinal tract is likely involved in a greater number of actions than previously thought, and instead it may be more useful to consider the relative degree of reticulospinal contribution, depending on the type of musculature involved and how the response is initiated. Specifically, the presentation of a loud (> 120 dB) startling acoustic stimulus (SAS) that elicits a startle reflex^3^ in a simple reaction time (RT) task leads to a dramatically shorter response latency in certain movement types (e.g. < 80 ms for arm or wrist extension movements). This RT reduction, known as the StartReact effect^4,5^, has been attributed to increased activation in reticular structures that mediate the startle reflex^6,7^ and thus an increased role of reticulospinal pathways in response initiation, which has recently been directly confirmed in primate research^8^.

The use of a StartReact protocol provides a relatively non-invasive approach to examine the reticulospinal contributions to a particular response. Although response latency is decreased when reacting to a startling stimulus versus the non-startling go signal, a key comparison examines the RT between SAS trials in which there is detectable activation in a startle reflex indicator muscle such as sternocleidomastoid (SCM), versus that on SAS trials in which there is no startle-related burst of activity. This ensures response latencies are compared between trials that all involve a high intensity go signal, and thus shorter RT latencies cannot simply be attributable to the well-known stimulus intensity effects whereby louder go signals result in shorter RT^9^. Instead, the only difference between these trial types is the confirmation of startle-related activation in the reticular formation, which is thought to be involved in the StartReact effect. Most StartReact studies have found significantly shorter RT for SCM + versus SCM− trials^10,11^, although one notable exception is unimanual finger abduction movements, which have repeatedly been shown to exhibit similar response latencies for SCM +/− trials^12–14^. The explanation given for this lack of a StartReact effect in the finger is that movements of the intrinsic hand muscles rely almost exclusively on corticospinal tracts, and thus the increased reticulospinal drive associated with elicitation of the startle reflex has little to no effect. However, individual finger responses can show a StartReact effect if modified in such a way that reticulospinal activation for the movement is increased, such as providing extensive practice^15^, pairing the response with an arm flexion movement^16^, or making the finger response bimanual rather than unimanual^14^.

These previous studies have suggested that the presence of a StartReact effect, as indexed by a significant RT difference between SCM + and SCM − trials, can be used to examine whether a specific response involves reticulospinal drive during initiation. However, response production for most movements likely involves a combination of various pathways and it is unknown if the StartReact protocol can be used to assess the *relative degree* of reticulospinal involvement. If a significant RT difference between SCM +/− trials is indicative of reticulospinal involvement for a given response, the magnitude of this difference may reflect the degree to which these pathways contribute to the movement. Being able to quantify the involvement of the reticulospinal pathway in an important area of exploration to understand how movements are performed, and may also provide additional information regarding its potential as a pathway to mediate functional recovery following stroke or spinal cord injury^17,18^.

The purpose of the current study was to examine the relative magnitude of the StartReact effect for two responses which are thought to involve different degrees of reticulospinal output. Although unimanual finger abduction does not show a StartReact effect^12,13^, bimanual finger responses show a small but significant (~ 10 ms) SCM +/− difference^14^ which has been attributed to increased reticulospinal input due to the bilateral nature of the movement. Conversely, proximal upper limb muscles such as those used to bilaterally abduct the shoulder are considered to be more strongly mediated by reticulospinal pathways^1^. Because these responses are thought to involve substantial differences in reticulospinal drive, it was hypothesized that a bilateral shoulder abduction movement would exhibit a larger RT difference between SCM +/− trials, as compared to a bimanual finger abduction movement. If so, these results would provide additional support for differential reticulospinal involvement for different types of movements as well as confirmation that a StartReact protocol can provide a simple and non-invasive method to investigate the relative contributions of different neural pathways involved in movement production. In addition to the expected RT differences, it was also of interest as to whether the SAS would differentially affect the EMG characteristics of both the startle reflex and prepared response for finger versus shoulder movements. Because activation of a finger response is assumed to interact less with reticulospinal pathways relative to the shoulder movement, it is possible that the startle reflex response would also be less frequently elicited, and potentially decreased in amplitude for this movement. Similarly, a difference in reticulospinal contributions may affect the degree to which the SAS amplifies the agonist EMG activation, which has been previously reported in StartReact studies^19,20^.

## Results

### Premotor RT

**Figure 1:**
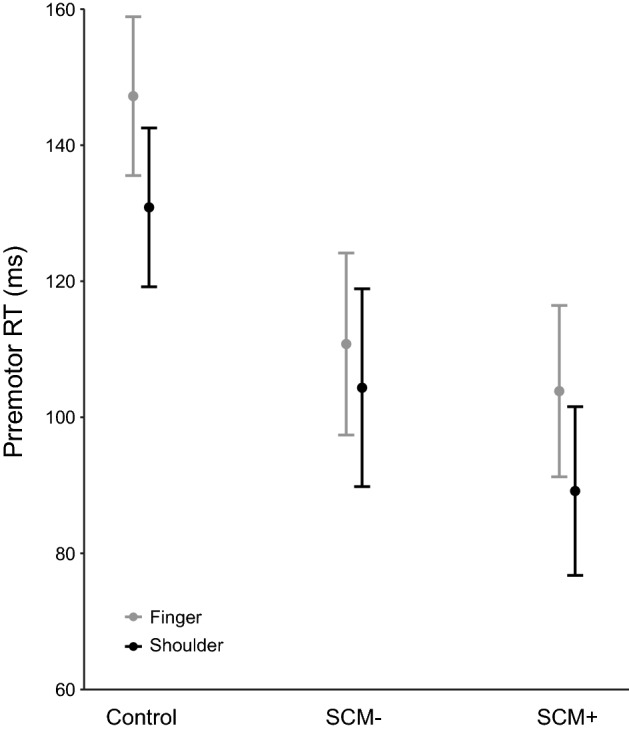
Premotor reaction time (RT; ms) shown for control trials, and startle trials in which activation in the sternocleidomastoid (SCM) was either absent (SCM −) or present (SCM +). Note the analysis only involved a comparison of SCM + /− conditions (n = 890 trials), whereby a significant interaction effect (p = 0.031) was found due to a larger decrease in RT for SCM + versus SCM − trials for the shoulder versus the finger response. Error bars represent 95% CI.

The analysis of premotor RT (n = 890 data points) revealed a significant main effect of stimulus, F(1, 881.6) = 35.4, p < 0.001 and task, F(1, 863.3) = 32.0, p < 0.001; however, these main effects were superseded by a significant interaction between the factors, F(1, 866.5) = 4.7, p = 0.031. Post-hoc analyses confirmed that although the SCM +/− difference was significant for both the shoulder task (SCM + mean = 88.7 ms, 95% CI [79.1, 98.2]; SCM − mean = 106.0 ms, 95% CI [95.5, 116.6]; p < 0.001) and finger task (SCM mean = 103.3 ms, 95% CI [93.6, 113.0]; SCM − mean = 112.4 ms, 95% CI [102.3, 122.5]; p = 0.006) the SCM +/− difference was significantly larger for the bilateral shoulder task (17.4 ms, SE = 3.1 ms) than for the bimanual finger task (9.1 ms, SE = 2.8 ms) (Fig. 1).

### Task-related EMG variables

**Figure 2:**
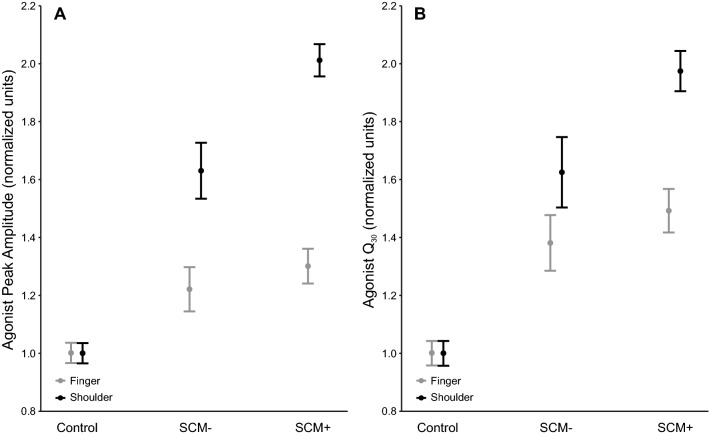
Task-related EMG variables (n = 3793 data points), separated by control trials and startle trials in which activation in the sternocleidomastoid (SCM) was either absent (SCM −) or present (SCM +). Panel (**A**) shows peak agonist amplitude (normalized to control values) whereas panel (**B**) shows normalized agonist Q_30_. Significant interaction effects were found for both variables (p < 0.001), due to significant differences between all conditions except for SCM + versus SCM − for the finger task and between control conditions. Error bars represent 95% CI.

The analysis of peak agonist amplitude (n = 3793 data points) showed a main effect for stimulus, F(2, 3763.3) = 558.5, p < 0.001 and task, F(1, 3771.3) = 240.1, p < 0.001, superseded by a significant Stimulus x Task interaction effect, F(2, 3780.1) = 160.5, p < 0.001 (Fig. 2A). Post-hoc analyses confirmed all pairwise comparisons were significantly different (all p-values < 0.001) except for SCM + and SCM − conditions for the finger task (p = 0.523), and between normalized control conditions (p = 1). Similar results were found for agonist Q_30_ (n = 3793 data points) with a significant main effect of stimulus, F(2, 3750.1) = 440.3, p < 0.001 and task, F(1, 3772.3) = 240.1, p < 0.001, but these effects were superseded by a significant Stimulus x Task interaction effect, F(2, 3780.1) = 160.5, p < 0.001 (Fig. 2B). Post-hoc analyses confirmed all pairwise comparisons of interest were significantly different (all p-values < 0.016) except for SCM + and SCM − conditions for the finger task (p = 0.413), and between normalized control conditions (p = 1).

### Startle reflex-related EMG variables

**Figure 3:**
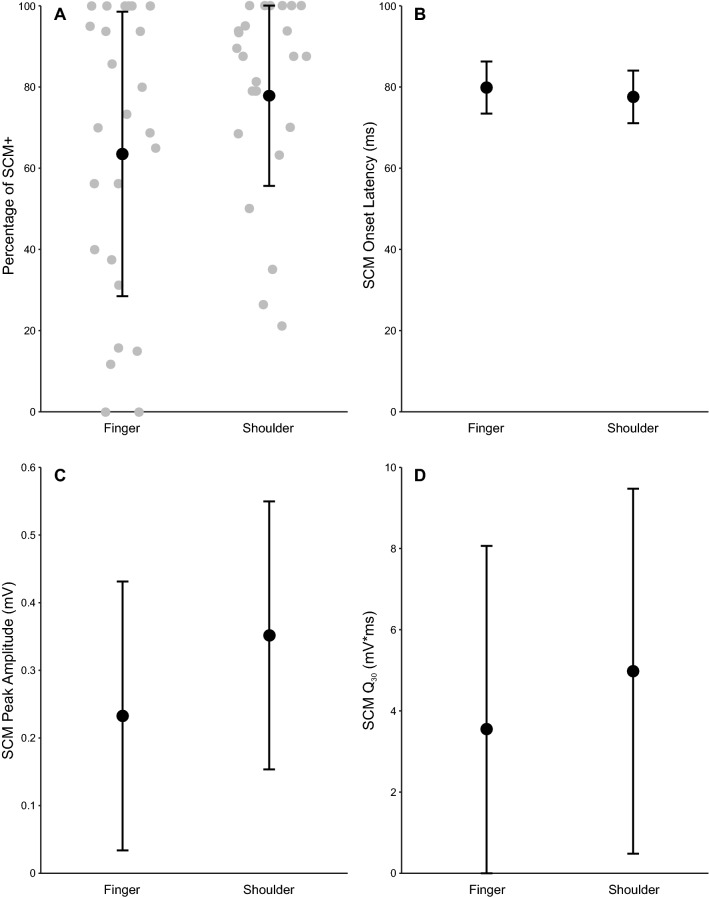
Startle-related EMG variables for trials in which sternocleidomastoid activation was present (SCM +) for the shoulder and finger responses. Panel (**A**) shows the proportion of SCM + trials, which was significantly higher for the shoulder task (p = 0.003) (n = 25 participants). Panel (**B**) shows the SCM onset latency in ms (n = 554 data points), which was not significantly different between movement types (p = 0.058). Panels (**C**) and (**D**) (n = 554 data points each) show SCM peak amplitude (mV) and SCM Q_30_ (mV*ms), which were both larger for the shoulder versus the finger task (p < 0.001 for peak amplitude, p = 0.016 for Q_30_). Error bars in panel (**A**) represent SD and grey dots show individual participant means; for all other panels error bars represent 95% CI.

The proportion of SAS trials that resulted in an observable startle reflex (i.e. SCM + trials) are shown in Fig. 3A (n = 25 participants). Analysis of these data showed a significant difference between the tasks, t(24) = 2.820, bootstrapped p = 0.003, d = 0.564, such that there was a larger proportion of SAS trials that elicited a startle reflex for the shoulder (mean = 79.2%, SD = 23.5%) than the finger (mean = 63.6%, SD = 35.1%). Mean difference = 15.6%, bootstrapped 95% CI [4.2, 27.1]. However, no significant effect of task was found for SCM onset (n = 554 data points; Fig. 3B), F(1, 550.2) = 3.62, p = 0.058, with similar latencies observed for the finger (mean = 77.5 ms, 95% CI [70.7, 84.3]) and shoulder (mean = 79.8 ms, 95% CI [73.0, 86.6]). For the SCM + trials, there was a significant effect of task on both SCM peak amplitude, F(1, 548.9) = 27.2, p < 0.001, (n = 554 data points; Fig. 3C) and SCM Q_30,_ F(1, 549.0) = 5.9, p = 0.016, (n = 554 data points; Fig. 3D), whereby the startle-related EMG characteristics were larger for the shoulder (mean peak amplitude = 0.352 mV, 95% CI [0.143, 0.561]; mean Q_30_ = 4.93 mV*ms, 95% CI [0.24, 9.62]) as compared to the finger (mean peak amplitude = 0.233 mV, 95% CI [0.023, 0.442]; mean Q_30_ = 3.92, 95% CI [− 1.19, 8.22]).

## Discussion

The purpose of the current study was to determine if the magnitude of the StartReact effect could be used as an indicator of the relative amount of reticulospinal output for a given movement. A proximal bilateral shoulder abduction task, which is thought to more strongly depend on reticulospinal drive, was contrasted with a distal bimanual finger abduction task, which is thought to have much weaker reticulospinal drive. Task-related and startle-related EMG characteristics were compared on SAS trials in which reflexive startle activation did (SCM +) or did not (SCM −) occur. These trials were selected as they both involved the high intensity go-signal, with the only difference being whether or not there was confirmation of startle-related activation in reticular structures following the SAS. As predicted, both tasks showed significantly shorter premotor RT latencies for SCM + as compared to SCM − trials; however, this difference was significantly larger for the shoulder task as compared to the finger task (Fig. 1). In addition, the influence of a startle on the amplitude of the task-related EMG response was different between tasks, whereby the agonist peak amplitude and Q_30_ were significantly larger for SCM + versus SCM − trials, but only for the more reticular-driven shoulder movement (Fig. 2). Lastly, task differences were also found for the startle reflex-related EMG characteristics, whereby the requirement to produce a bilateral shoulder response as compared to a finger response, resulted in a significantly higher proportion of trials with an observed startle reflex in SCM. Furthermore, the SCM response seen in these trials was produced with increased peak SCM amplitude and SCM Q_30_ (Fig. 3). Collectively, these results provide additional confirmation of differential reticulospinal involvement for proximal versus distal movements, and also indicate that StartReact can be used as a novel and non-invasive indirect method of quantifying the relative contributions of neural pathways involved in response initiation.

The use of a StartReact protocol has become a more common and useful method to investigate the processes involved in response preparation and their associated timelines^4^. The most common explanation for the large RT reduction seen on SAS trials is that increased activation in reticular structures associated with the startle reflex results in a faster accumulation of initiation-related activation, which in turn leads to the response being triggered at a much shorter latency^21^. However, simply comparing response latencies on control and startle trials does not fully allow for investigation of the StartReact effect as auditory trials can also be affected by intersensory facilitation and stimulus intensity^22,23^. Instead, a comparison between SAS trials in which startle-related activation is present versus absent provides a more rigorous control for these confounding effects. Indeed, when a loud acoustic stimulus also elicits a startle reflex (i.e. SCM +), which is an indication of activation in the reticular formation, RT reduction and the incidence of response triggering are increased relative to when no startle reflex is elicited (SCM −)^24–26^. While this does not mean that SCM − trials are devoid of reticulospinal activation, these previous results clearly show that the presence of SCM activation leads to a more robust StartReact effect that is unbiased by stimulus intensity. The most parsimonious explanation for these effects is that greater reticulospinal drive is present on SCM + trials and thus a comparison between SCM +/− trials can be used as a surrogate measure of relative reticulospinal contributions to the response. This assertion is supported by studies examining the magnitude of the StartReact effect for different responses. For example, unimanual finger movements do not show differences between SCM + and SCM − trials^12–14^ and the explanation for this reduced impact of startle is that intrinsic hand muscles are thought to be heavily dependent upon corticospinal drive, and as such any impact of increased reticulospinal drive is minimal. Additionally, reticulospinal drive is thought to be increased during bilateral responses^27,28^, which would be expected to manifest as a small SCM +/− difference for bimanual finger responses, as has been shown previously^14^, as well as in the current study. Similarly, proximal responses involved in postural control are thought to involve greater reticulospinal drive than distal responses^1^ and thus a bilateral shoulder response would be expected to show a more substantial SCM +/− difference than a bimanual finger response, also found in the current study (Fig. 1).

In addition to the differences in response latency, the current study also found differences in how the SAS affected the task-related EMG amplitude. Some previous StartReact studies have reported increased agonist EMG amplitude on startle trials, which has been attributed to additional motor output following a more intense go-signal^19,20^. The current study allowed for a unique evaluation of whether these effects are due to stimulus intensity, startle reflex activation, or both. As expected, presentation of the SAS resulted in increases in task-related EMG amplitude (relative to control trials) on SCM + and SCM − trials for both movements (Fig. 2). However, only the more reticular dependent shoulder movement showed an increase in task-related EMG amplitude *between* SCM + and SCM − trials. Furthermore, this normalized increase in EMG amplitude on all SAS trials (SCM + and SCM −) was much larger overall for the proximal shoulder task. This novel finding indicates that when a startle reflex is elicited on SAS trials, the increased reticulospinal drive can result in a larger task-related EMG output. Interestingly, this effect was only seen for the shoulder task, which supports the hypothesis that proximal muscles receive greater reticulospinal input. In addition, a smaller increase in task-related EMG amplitude was seen on SCM − trials, suggesting that a more intense stimulus can result in increased response output, even when the startle reflex is not elicited. The ability to increase the strength of response output via the engagement of reticular-mediated pathways has important implications. For example, a SAS may be used as a tool to improve functional movement amplitude in individuals with stroke, leading to responses that are more similar to those produced by unimpaired control participants^29,30^.

While the present results supported the hypothesis that muscles presumed to receive increased reticulospinal drive would show a larger StartReact effect and increased response output magnitude, the startle-reflex data further suggest that this relationship may be bidirectional. Specifically, the proportion of trials in which SCM activation was observed, as well as measures of startle-reflex amplitude, were larger in the shoulder versus finger movement (Fig. 3). This result can be explained by hypothesizing that increased activation in reticular structures occurs during preparation of a proximal versus distal task, thus making it more likely to elicit a startle reflex, which would be larger in amplitude. On the other hand, the SCM onset was not affected by the type of task (Fig. 3B), suggesting that *if* startle-related SCM activation is observed, it nevertheless occurs at a similar latency.

It is worthwhile to consider some alternative explanations for the current results. One alternative hypothesis has attributed the RT difference between SCM + and SCM − trials to differences in response preparation levels, such that SCM − is more likely to occur on trials where lower preparatory activation is achieved^31^, resulting in a longer RT latency, a decreased likelihood of eliciting a startle reflex, and a smaller EMG amplitude due to reduced H-reflex gain. Although response preparation is known to affect response latency in a simple RT task, it does not appear that preparation level differences systematically underlie the probability of obtaining SCM +/− trials. Specifically, previous work has shown that motor preparation levels indexed by TMS-induced motor evoked potentials are statistically equivalent between SCM + and SCM − trials, suggesting that RT differences between SCM +/− trials are not a function of response preparation^32^. The increase in EMG amplitude and shortening of RT observed on SCM + trials could alternatively be attributed to superimposed startle-related and/or audiospinal reflex-related activation onto the voluntary response, rather than early triggering of the prepared response alone. This explanation has been extensively explored and rejected due to the preservation of the EMG timing characteristics of the prepared response on startle trials^7,33^. Lastly, the current data show between-task differences in control RT latencies (Fig. 1) that were not accounted for in the analysis. However, the key comparison in the present research question involved an examination of how the different tasks led to differences in the dependent measures between SCM + and SCM − trials, because these conditions both involved a high intensity go-stimulus and thus the only difference was the presence/absence of startle reflex-related EMG activation. It may be that nerve conduction time differences contributed to the observed results due to the longer distance to reach the finger versus shoulder muscles, but if conduction time were the primary driver of this effect, it would be expected to present across all trials including SCM − trials. As the more proximal muscle showed a greater RT reduction on SCM + trials, as well as an increase in both agonist and SCM EMG measure, we believe these task differences are best explained by an increase in the reticulospinal drive associated with the shoulder task.

Collectively, the results from the current study provide strong and novel evidence for differential contributions from reticulospinal structures to proximal versus distal effectors in humans. Understanding that the neural pathways for specific voluntary movements involve different amounts of cortical and reticular drive may have a wide variety of important applications ranging from stroke rehabilitation to prosthetic limb control. In addition, for the first time these results show a bidirectional relationship between task preparation and the startle reflex. In this way, the startle reflex is more likely to be observed and with greater amplitude, when preparing a proximal versus distal task.

## Materials and methods

### Participants

Twenty-five participants that self-reported as right-handed or ambidextrous, with normal or corrected to normal vision and no neurological or motor impairments, participated in the study (15 M, 10F; mean age 24.7 years, SD = 8.2). All volunteers provided written informed consent prior to taking part. The experimental protocol was approved by and conducted in accordance with the ethical guidelines of the University of Ottawa Health Sciences and Science Research Ethics Board (H04-16-01) and conformed to the most recent revision of the Declaration of Helsinki.

### Apparatus and task

Participants sat comfortably in a height adjustable chair with arms unrestricted approximately 1.5 m away from a 24” LCD computer monitor (Asus VG248; 144 Hz refresh) and completed two simple RT tasks presented in sequential blocks (task order counterbalanced across participants). The first task involved a rapid simultaneous bimanual contraction of the first dorsal interosseous (FDI) muscles resulting in abduction of only the index fingers (approximately 15°) while the hands were comfortably resting with palms inward on the participant’s lap. The third, fourth, and fifth digits of each hand were relaxed but curled as if grasping an imaginary post, with the second digit extending directly forward resting on the third digit. In the second task, participants held their arms along the sides of the body with the elbows flexed at 90° and palms inward, and were required to perform a rapid simultaneous bilateral contraction of the lateral deltoid (LD) resulting in upper arm abduction (approximately 30°). Participants were instructed to react by making the required bilateral movement as quickly and accurately as possible following an acoustic go-signal, with the emphasis placed on producing fast movements. Each type of task began with 10 practice trials (no SAS) to allow participants to become familiar with the movement, followed by four blocks of 20 experimental trials. Feedback regarding RT and limb position (accuracy) were provided on the computer screen at the end of each trial for 3.5 s. On each trial, RT was provided and points were awarded for RTs faster than 140 ms (1 point per ms to a maximum of 25 points per trial). These points had no monetary significance and were simply used to increase participant motivation to perform accurate movements initiated at short latency. If RT was shorter than 40 ms, a message appeared following trial completion instructing the participant to wait for the go-signal. Testing lasted approximately 1 h and participants were given the option to rest after each block in order to minimize fatigue and to maintain focus on the task.

### Instrumentation and stimuli

In each trial, an acoustic warning signal (80 dB, 200 Hz, 100 ms) was followed at variable latency (2000–2500 ms) by an auditory go-signal (82 dB, 1000 Hz, 40 ms). On 20% of experimental trials, a SAS (120 dB, white noise, 25 ms) unexpectedly replaced the go-signal, such that each testing block of 20 trials consisted of 16 non-startle and 4 SAS trials. Analog signals were generated using digital to analog hardware (PCIe-6321, National Instruments Inc., Austin, TX) and were amplified and presented via a loudspeaker (M54-H, MG Electronics, Inc., Hauppauge, NY) placed 30 cm directly behind the participant’s head. Stimulus intensity was confirmed using a precision sound level meter (Cirrus Research Optimus, CR:162C; A-weighted, impulse setting) placed at the location of the participant’s left ear during testing. SAS trials were pseudorandomized, such that no block began with a SAS trial and no two sequential trials involved a SAS.

Index finger displacement was calculated by double integrating acceleration data collected from a single axis piezoelectric accelerometer (Type 4375, Brüel and Kjær) attached to the lateral aspect of the right index finger (distal to the first distal interphalangeal joint) using tape. The accelerometer was connected through an inline charge amplifier (Type 422, PCB Electronics) to a power supply and coupler (Model 5114, Kistler Inc.) with real-time analog output. Shoulder joint angle was measured using an electrogoniometer (Biometrics B1707) with one end secured to the lateral aspect of the right upper arm, and the other end secured to the posterior aspect of the upper right trapezius using double sided tape. The goniometer was connected to a data acquisition unit (DataLink DLK900, Biometrics Ltd., Gwent, UK) with real-time analog output. Surface EMG data were collected bilaterally from the muscle bellies of the LD and FDI muscles (prime mover/agonist for each task, respectively), as well as bilaterally from sternocleidomastoid (SCM) muscles (to confirm presence of startle reflex) using bipolar preamplified surface electrodes (Delsys Bagnoli DE-3.1; Delsys Inc., Natick, MA) connected to an external amplifier system (Delsys Bagnoli-8) via shielded cabling. EMG electrodes were placed parallel to the orientation of the muscle fibres. Attachment sites were cleaned using abrasive gel and an alcohol swab in order to minimize impedance, and electrodes were attached using two-sided adhesive tape. Detrended raw accelerometer and goniometer output, as well as band-pass filtered (20–450 Hz) EMG signals were digitally sampled at 1000 Hz (PCIe-6321, National Instruments Inc.) for 3 s starting 1 s prior to the go-signal on each trial.

### Data reduction

EMG parameters were determined from the raw data using a custom LabVIEW analysis program, in which EMG data for each trial were full‐wave rectified and dual-pass filtered using a 25 Hz low‐pass second‐order elliptic filter. EMG onset was initially determined as the first point where the rectified and filtered EMG first reached a point greater than two standard deviations above the baseline noise (determined from a window of 100 ms of activity preceding the go-signal) and remained elevated for at least 20 ms. EMG offset was determined as the first point after peak EMG where EMG fell below 20% of the maximal value for the burst. These points were then visually inspected and manually adjusted whenever necessary.

The primary dependent variable was premotor RT, which was defined as the time from the go-signal/SAS to EMG onset in the prime mover. In addition, task-related EMG amplitude measures included Q_30_^34,35^, which was calculated as a numeric integration of the first 30 ms of rectified raw EMG following burst EMG onset, and peak agonist amplitude which was calculated as the largest EMG amplitude recorded within an interval of 100 ms following EMG burst onset (calculated from rectified and filtered EMG). Task-related EMG amplitude measures for each trial were normalized as a proportion of each participant’s grand mean amplitude obtained from that muscle in all control (non-SAS) trials. Movement kinematics were not analyzed as the accelerometer and goniometer did not provide consistently reliable outputs, and therefore were primarily used to provide approximate limb displacement feedback to the participant.

Startle trials were designated as SCM + if a burst of EMG activity in either the left or right SCM was observed between 25 and 120 ms following the SAS, a time range where startle-related SCM EMG activity is likely to occur^3,36^; otherwise, SAS trials were classified as SCM −. Startle reflex-related EMG variables including SCM onset latency, SCM Q_30_ and peak SCM amplitude (as described above for the agonist muscle) were examined to determine if the characteristics of the startle reflex differed depending on the movement task for a given trial.

Practice trials were not included in the analysis, nor were trials with RT < 50 ms (anticipation, 100 trials), RT > 400 ms (inattentiveness, 50 trials), trials where movement error occurred (e.g., single limb movement, 14 trials), or trials with an EMG onset asynchrony (> 50 ms asynchrony, 43 trials). Overall, these procedures led to the retention of 3793/4000 trials (94.8%).

### Statistical analysis

Where appropriate, data were analyzed using linear mixed effects (LME) analyses, as they allow all data points to be retained (i.e. multiple trials from any condition for the same participant can be included in the analysis) without violating assumptions of independence^37^. All variables were analyzed from the right side only, as asynchronous trials were excluded from analysis. For premotor RT, control trials were not included in the analysis, as it was expected that stimulus intensity effects would result in a substantial reduction in RT on startle trials, and the difference between SCM + versus SCM − trials was the critical comparison to answer our research question. Furthermore, inclusion of control trial data and its associated variance (heteroscedasticity) could mask any small effects of SCM presence on the variables. Thus, premotor RT were analyzed using an LME model whereby SCM presence (SCM +, SCM −) and Task (finger, shoulder) were specified as interacting fixed factors, and intercepts for subjects were specified as a random effect (e.g. [model = premotor RT ~ SCM * Task + (1| Subject)]). The task-related EMG amplitude measures (agonist Q_30_ and peak agonist amplitude) were each analyzed using an LME model whereby Condition (control, SCM +, SCM −) and Task (finger, shoulder) were specified as interacting fixed factors, and intercepts for subjects were specified as a random effect. The proportion of SAS trials that led to an observed burst of EMG activity in SCM was analyzed between the bimanual finger and bilateral shoulder task conditions using a bootstrapped paired samples t-test. In addition, for SCM + trials only, the startle reflex EMG characteristics (SCM onset, SCM Q_30_ and peak SCM amplitude) were each analyzed using an LME model whereby Task (finger, shoulder) was specified as a fixed factor, and intercepts for subjects were specified as a random effect.

The significance value for all statistical tests was set at p < 0.05. Data used in LME models was examined for homoscedasticity and approximate normal distribution of residuals as well as scanned for influential cases. LME analyses were performed with R statistical software^38^ using the lme4 package^39^ along with the lmerTest package^40^ to provide p-values. Planned pairwise contrasts were conducted in R using the emmeans package^41^ with Tukey’s correction for multiple comparisons. Note that measures of variability for the LME analyses are provided using 95% CI values, because this type of analysis does not provide variance estimates using standard deviations, and thus “n” values represent the total number of data points analyzed across participants. Cohen’s d values are provided as a measure of effect size for t-tests.

## Data Availability

The datasets generated during and analysed during the current study are available from the corresponding author on reasonable request.

## References

[1] Prentice SD, Drew T (2001). Contributions of the reticulospinal system to the postural adjustments occurring during voluntary gait modifications. J. Neurophysiol..

[2] Schepens B, Drew T (2004). Independent and convergent signals from the pontomedullary reticular formation contribute to the control of posture and movement during reaching in the cat. J. Neurophysiol..

[3] Brown P (1991). New observations on the normal auditory startle reflex in man. Brain.

[4] Carlsen AN, Maslovat D, Franks IM (2012). Preparation for voluntary movement in healthy and clinical populations: Evidence from startle. Clin. Neurophysiol..

[5] Valls-Solé J, Kumru H, Kofler M (2008). Interaction between startle and voluntary reactions in humans. Exp. Brain Res..

[6] Carlsen AN, Chua R, Inglis JT, Sanderson DJ, Franks IM (2004). Can prepared responses be stored subcortically?. Exp. Brain Res..

[7] Valls-Solé J, Rothwell JC, Goulart F, Cossu G, Munoz E (1999). Patterned ballistic movements triggered by a startle in healthy humans. J. Physiol..

[8] Tapia JA, Tohyama T, Poll A, Baker SN (2022). The existence of the StartReact effect implies reticulospinal, not corticospinal, inputs dominate drive to motoneurons during voluntary movement. J. Neurosci..

[9] Woodworth RS (1938). Experimental Psychology.

[10] Leow LA (2018). Triggering mechanisms for motor actions: The effects of expectation on reaction times to intense acoustic stimuli. Neuroscience.

[11] McInnes AN (2021). Cumulative distribution functions: An alternative approach to examine the triggering of prepared motor actions in the StartReact effect. Eur. J. Neurosci..

[12] Carlsen AN, Chua R, Inglis JT, Sanderson DJ, Franks IM (2009). Differential effects of startle on reaction time for finger and arm movements. J. Neurophysiol..

[13] Honeycutt CF, Kharouta M, Perreault EJ (2013). Evidence for reticulospinal contributions to coordinated finger movements in humans. J. Neurophysiol..

[14] Maslovat D, Teku F, Smith V, Drummond NM, Carlsen AN (2020). Bimanual but not unimanual finger movements are triggered by a startling acoustic stimulus: Evidence for increased reticulospinal drive for bimanual responses. J. Neurophysiol..

[15] Kirkpatrick NJ, Ravichandran VJ, Perreault EJ, Schaefer SY, Honeycutt CF (2018). Evidence for startle as a measurable behavioral indicator of motor learning. PLoS ONE.

[16] Castellote JM, Kofler M (2018). StartReact effects in first dorsal interosseous muscle are absent in a pinch task, but present when combined with elbow flexion. PLoS ONE.

[17] Baker SN, Zaaimi B, Fisher KM, Edgley SA, Soteropoulos DS (2015). Pathways mediating functional recovery. Prog. Brain Res..

[18] Zaaimi B, Edgley SA, Soteropoulos DS, Baker SN (2012). Changes in descending motor pathway connectivity after corticospinal tract lesion in macaque monkey. Brain.

[19] Maslovat D, Hodges NJ, Chua R, Franks IM (2011). Motor preparation of spatially and temporally defined movements: Evidence from startle. J. Neurophysiol..

[20] Maslovat D, Franks IM, Leguerrier A, Carlsen AN (2015). Responses to startling acoustic stimuli indicate that movement-related activation is constant prior to action: A replication with an alternate interpretation. Physiol. Rep..

[21] Carlsen AN, Maslovat D (2019). Startle and the StartReact effect: Physiological mechanisms. J. Clin. Neurophysiol..

[22] Kohfeld DL (1971). Simple reaction time as a function of stimulus intensity in decibels of light and sound. J. Exp. Psychol..

[23] Nickerson RS (1973). Intersensory facilitation of reaction time: Energy summation or preparation enhancement?. Psychol. Rev..

[24] Maslovat D, Sadler CM, Smith V, Bui A, Carlsen AN (2021). Response triggering by an acoustic stimulus increases with stimulus intensity and is best predicted by startle reflex activation. Sci. Rep..

[25] Carlsen AN, Dakin CJ, Chua R, Franks IM (2007). Startle produces early response latencies that are distinct from stimulus intensity effects. Exp. Brain Res..

[26] Carlsen AN (2015). A broadband acoustic stimulus is more likely than a pure tone to elicit a startle reflex and prepared movements. Physiol. Rep..

[27] Davidson AG, Chan V, O’Dell R, Schieber MH (2007). Rapid changes in throughput from single motor cortex neurons to muscle activity. Science.

[28] Davidson AG, Buford JA (2006). Bilateral actions of the reticulospinal tract on arm and shoulder muscles in the monkey: Stimulus triggered averaging. Exp. Brain Res..

[29] Lee H, Honeycutt C, Perreault E (2022). Influence of task complexity on movement planning and release after stroke: Insights from startReact. Exp. Brain Res..

[30] Rahimi M, Honeycutt CF (2020). StartReact increases the probability of muscle activity and distance in severe/moderate stroke survivors during two-dimensional reaching task. Exp. Brain Res..

[31] Marinovic W, Tresilian JR (2016). Triggering prepared actions by sudden sounds: Reassessing the evidence for a single mechanism. Acta Physiol..

[32] Smith V, Maslovat D, Carlsen AN (2019). StartReact effects are dependent on engagement of startle reflex circuits: Support for a subcortically mediated initiation pathway. J. Neurophysiol..

[33] Carlsen AN, Chua R, Inglis JT, Sanderson DJ, Franks IM (2004). Prepared movements are elicited early by startle. J. Motor Behav..

[34] Corcos DM, Gottlieb GL, Agarwal GC (1989). Organizing principles for single-joint movements. II. A speed-sensitive strategy. J. Neurophysiol..

[35] Gottlieb GL, Corcos DM, Agarwal GC (1989). Organizing principles for single-joint movements. I. A speed-insensitive strategy. J. Neurophysiol..

[36] Carlsen AN, Maslovat D, Lam MY, Chua R, Franks IM (2011). Considerations for the use of a startling acoustic stimulus in studies of motor preparation in humans. Neurosci. Biobehav. Rev..

[37] Magezi DA (2015). Linear mixed-effects models for within-participant psychology experiments: An introductory tutorial and free, graphical user interface (LMMgui). Front. Psychol..

[38] R Core Team. R: A language and environment for statistical computing. *Foundation for Statistical Computing* (2019).

[39] Bates D, Machler M, Bolker BM, Walker SC (2015). Fitting linear mixed-effects models using lme4. J. Stat. Softw..

[40] Kuznetsova A, Brockhoff PB, Christensen RHB (2017). lmerTest package: Tests in linear mixed effects models. J. Stat. Softw..

[41] Lenth RV (2016). Least-squares means: The R package lsmeans. J. Stat. Softw..

